# Birth control: to what extent do women report being informed and involved in decisions about pregnancy and birth procedures?

**DOI:** 10.1186/1471-2393-14-62

**Published:** 2014-02-07

**Authors:** Rachel Thompson, Yvette D Miller

**Affiliations:** 1 School of Psychology, The University of Queensland, Brisbane, QLD 4072, Australia; 2 The Dartmouth Center for Health Care Delivery Science, Dartmouth College, Hanover, NH 03755, USA; 3 School of Public Health and Social Work, Queensland University of Technology, Kelvin Grove, QLD 4059, Australia

**Keywords:** Decision-making, Maternity care, Paternalism, Patient participation, Patient-reported outcomes

## Abstract

**Background:**

Health policy, guidelines, and standards advocate giving patients comprehensive information and facilitating their involvement in health-related decision-making. Routine assessment of patient reports of these processes is needed. Our objective was to examine decision-making processes, specifically information provision and consumer involvement in decision-making, for nine pregnancy, labour, and birth procedures, as reported by maternity care consumers in Queensland, Australia.

**Methods:**

Participants were women who had a live birth in Queensland in a specified time period and were not found to have had a baby that died since birth, who completed the extended *Having a Baby in Queensland Survey, 2010* about their maternity care experiences, and who reported at least one of the nine procedures of interest. For each procedure, women answered two questions that measured perceived (i) receipt of information about the benefits and risks of the procedure and (ii) role in decision-making about the procedure.

**Results:**

In all, 3,542 eligible women (34.2%) completed the survey. Between 4% (for pre-labour caesarean section) and 60% (for vaginal examination) of women reported not being informed of the benefits and risks of the procedure they experienced. Between 2% (epidural) and 34% (episiotomy) of women reported being unconsulted in decision-making. Over one quarter (26%) of the women who experienced episiotomy reported being neither informed nor consulted.

**Conclusions:**

There is an urgent need for interventions that facilitate information provision and consumer involvement in decision-making about several perinatal procedures, especially those performed within the time-limited intrapartum care episode.

## Background

The paternalistic model of treatment decision-making, characterised by a care provider taking the active role in treatment decision-making and a passive and acquiescent patient, has been challenged in recent years in favour of alternative doctor-patient partnership models [1]. The informed decision-making model is one such alternative, characterised by the care provider communicating information on all relevant treatment options and their benefits and risks to a patient, and the patient deciding on a treatment option [1]. The shared decision-making model is another, where there is mutual exchange of information by provider and patient and joint deliberation and decision-making about the treatment option to implement [1,2].

Over the past two decades, health policy and legislation, clinical guidelines, and professional standards in several countries have increasingly emphasised patient participation in decision-making, alongside comprehensive information provision. This emphasis is particularly evident in the maternity care sector. In the United Kingdom, the landmark *Changing Childbirth* report, published in 1993, advocated for the provision of woman-centred maternity care that supports consumers to make informed choices and exercise autonomy and control [3], and several subsequent documents have reinforced this objective. In Australia, the *National Maternity Services Plan 2010* recommended that maternity care services should enable women to access objective, evidence-based information that supports them to make informed choices in accordance with their individual needs [4]. In the United States, a recent joint statement endorsed by seven maternity care professional organisations stated that “[d]ecisions about interventions should incorporate the woman’s personal values and preferences and should be made only after she has had enough information to make an informed choice, in partnership with her care team” [5].

This growing emphasis on patient participation in policy, guidelines, and standards is consistent with most consumers’ preferences. Although there is not a universal desire for decisional autonomy, most maternity care consumers wish to at least participate in decision-making. A survey of 1,336 new mothers in Australia found that over 96% had wanted to have an active say in decision-making during labour [6]. In Scotland, a study of 301 pregnant women at low obstetric risk found that the vast majority wanted either to control decision-making (48%) or to be involved (42%) [7]. Only 9% of women wanted to be informed but not involved in decision-making, and 1% wanted staff to make decisions for them [7]. A third study in Wales found that 90% of pregnant women and 83% of postnatal women preferred either to make final decisions themselves or to share decision-making with care providers [8].

Given the policy significance of information provision and patient participation in decision-making, and its importance to consumers, routine assessment of women’s experiences of these aspects of pregnancy, labour and birth care is needed to evaluate the quality of maternity services and inform quality improvement priorities. While there have been previous attempts to assess women’s involvement in pregnancy, labour and birth decision-making [8,9], the use of selective samples has limited the usefulness of findings for understanding care at a whole-of-system level. The few studies we could identify that have examined participation in decision-making either state- or country-wide have focused only on one or two specific procedures [10,11] or have studied decision-making processes globally for an entire episode of care [11,12]. One other population-level study of women’s maternity care experiences assessed receipt of information about four antenatal screening tests in the United Kingdom, but did not measure role in decision-making [13].

In this study, our objective was to examine decision-making processes for nine pregnancy, labour, and birth procedures, as reported by maternity care consumers in Queensland, Australia. Using data from a large, statewide survey, we analysed the prevalence of six different approaches to decision-making based on (i) the presence or absence of information provision about the benefits and risks of the procedure, and (ii) the woman’s role in decision-making. We describe patterns in decision-making processes across the nine procedures studied and implications for both research and maternity care improvement.

## Methods

### Participants and survey procedure

Participants in this study were respondents to the *Having a Baby in Queensland Survey, 2010*[14]. The sampling frame for this survey was databases of compulsory birth notification and registration records, held by the *Queensland Registry of Births, Deaths and Marriages*. All women who had a live birth in Queensland, Australia in a four-month period, and who were not found to have had a baby that died since birth, were eligible to be surveyed. Two versions of the survey were administered. A survey containing only core items (the basic survey) was administered to half of the women with a singleton birth in the sampling period, and is not discussed further here. The remaining half of the women with a singleton birth, and all of the women with a multiple birth in the sampling period, were administered a survey containing core and supplementary items (the extended survey).

The entire eligible population for the extended survey was sent a survey package four to five months after birth. The package included an English-language information sheet, an English-language paper survey, and participation instructions in 19 other languages (Cantonese, Mandarin, Greek, Korean, Persian, Russian, Serbian, Spanish, Turkish, Vietnamese, German, Arabic, French, Samoan, Filipino, Dinka, Japanese, Khmer and Amharic). Women could (i) complete and return the paper survey using a reply-paid envelope, (ii) complete the same survey online, or (iii) complete only core survey items via telephone (free call) with a female interviewer and, if necessary, a translator from the Australian Government *Translating and Interpreting Service*. All women were gifted a pen and those who completed the survey within a specified timeframe were invited to enter a draw to win one of four $200 gift cards. All women were sent a reminder to complete the survey approximately two weeks after the initial mailing.

The sample for the current study comprised those women who completed the extended survey and reported at least one of the following nine procedures: ultrasound scan, blood test, induction of labour, pre-labour caesarean section, vaginal examination, fetal monitoring during labour, post-labour caesarean section, epidural anaesthesia, and episiotomy.

### Measures

The *Having a Baby in Queensland Survey, 2010*[14] was developed by the authors to retrospectively assess consumers’ experiences of care during pregnancy, labour and birth, and after birth. The final survey instrument resulted from comprehensive reviews of similar surveys undertaken elsewhere, and extensive consultation with women, providers, and other stakeholders. Survey items relevant to the current analyses are detailed below.

#### Background and care characteristics

Women’s socio-demographic characteristics including age at birth, parity, highest level of education, indigenous identification and country of birth were assessed. Birth plurality was coded from the type of survey completed (based on birth notification records). The remoteness of participants’ area of residence was determined from postcode and suburb of usual residence according to the ARIA + classification of remoteness and accessibility [15], endorsed by the *Australian Bureau of Statistics*. Women’s place of birth was assessed using multiple items and was subsequently coded into five categories (private hospital, public hospital, public birth centre, home (planned) and other).

#### Pregnancy, labour and birth procedures

Women’s experiences of the nine pregnancy, labour and birth procedures – ultrasound scan(s) in pregnancy (for any reason), blood test(s) in pregnancy (for any reason), induction of labour, pre-labour caesarean section, vaginal examination during labour, fetal monitoring during labour, post-labour caesarean section, epidural anaesthesia during labour, and episiotomy during vaginal birth – were assessed. Women who had a multiple birth and experienced a vaginal birth for the first-born baby and a caesarean section for a subsequently born baby (n = 1) were not included in the subsample of women with a cesarean section.

#### Decision-making process

The decision-making process for each of the nine procedures was assessed via pairs of items that measured (i) receipt of information, and (ii) role in decision-making.

##### 

**(i) Receipt of information** To assess receipt of information, participants were asked to recall whether care providers discussed with them the outcomes associated with having the procedure, and not having the procedure. No timeframe was specified. Item wording was tailored for each procedure (e.g., “*Did your maternity care provider(s) discuss with you the pros and cons (benefits and risks) of having and not having a caesarean?*”) and a yes/no response option provided. Cognitive interviews were undertaken with several women in the process of survey development to maximise the understandability and validity of items prior to their use. Findings from these cognitive interviews suggested that participants did not find the double-barreled nature of these questions challenging.

##### 

**(ii) Role in decision-making** The single-item Control Preferences Scale, developed by Degner, Sloan and Venkatesh to assess preferred decisional role [16] and often used in modified form to measure *actual* decisional role [17-20], was further adapted to assess role in decision-making. Again, the item was tailored for each procedure (e.g., “*Who decided if you would or would not have a caesarean?*”). There were three response options: (i) “*I decided from all my available options*”, (ii) “*My maternity care provider(s) decided and checked if it was OK with me*”, and (iii) “*My maternity care provider(s) decided without checking with me*”. These three response options were reduced from the usual five response options by eliminating alternatives corresponding to the patient deciding after considering the provider’s opinion, and the patient and provider sharing decision-making, as we describe below.

Previous research has observed ceiling effects in the measurement of patient involvement in decision-making [21,22]. Cognisant of this, we removed the response option corresponding to shared decision-making to prevent women misclassifying consent to a procedure as shared decision-making. We anticipated that when faced with only the three alternatives, women who merely consented to a procedure would select “*My maternity care provider(s) decided and checked if it was OK with me”* and that women who genuinely participated in shared decision-making (as well as those who considered their providers’ opinions before deciding on a procedure) would select “*I decided…*”. Notably, we were not concerned with distinguishing between shared and patient-led decision-making. As noted above, cognitive interviews were undertaken with several women in the process of survey development. Findings from these cognitive interviews suggested that, although some interviewees found decisional role questions challenging, they ultimately selected responses that were aligned with the researchers’ intentions and assumptions.

### Ethical approval

Ethical approval for the *Having a Baby in Queensland Survey, 2010* and subsequent analyses was obtained from The University of Queensland *Behavioural & Social Sciences Ethical Review Committee* (Clearance #2010000613).

### Analytic strategy

A six-category composite variable representing decision-making process was derived for each of the nine procedures, using coding rules developed a priori (see Table 1). Nine (non-mutually-exclusive) samples were created to represent women who reported having experienced each of the nine procedures of interest. Using these samples, descriptive analyses were conducted to determine the prevalence of the different decision-making approaches for each procedure.

**Table 1:** Rules for coding decision-making processes

		**Role in decision-making:**		
		**Decided from all available options**	**Did not decide, checked with**	**Did not decide, not checked with**
**Receipt of information:**	**Yes**	‘Informed decided’	‘Informed consulted’	‘Informed unconsulted’
	**No**	‘Uninformed decided’	‘Uninformed consulted’	‘Uninformed unconsulted’

## Results

### Participant flowchart

**Figure 1:**
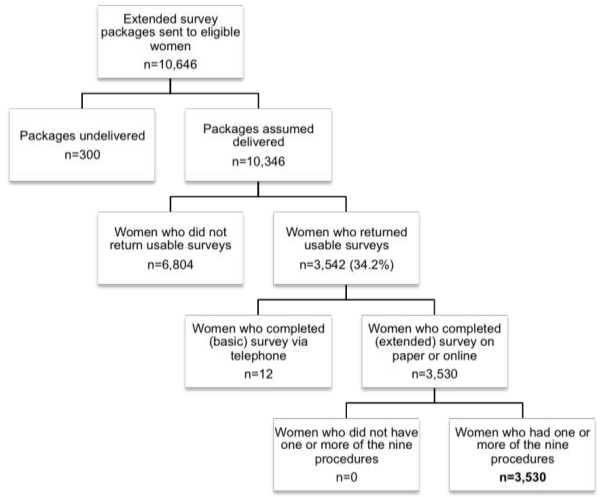
Flowchart of participants.

Of the 10,346 eligible women who were assumed to have received the extended survey, 3,542 (34.2%) responded, with 3,530 of these completing the extended survey on paper or online. All of these respondents experienced at least one of the procedures of interest (see Figure 1).

### Participant characteristics

Background and care characteristics of the sample, as well as of the Queensland birthing population, are provided in Table 2. The sample was diverse in age and remoteness of residence, and respondents were fairly evenly divided between primiparity (45%) and multiparity (55%). A large majority of women was born in Australia (80%), did not identify as Indigenous (98%), and had at least a secondary school education (90%). Most women had a singleton birth (97%) and birthed in a conventional hospital setting (97%).

**Table 2:** Background and care characteristics of survey respondents (n = 3530)

	**Sample**		Population*
	**Freq.**	**%**	**%**
Age at birth			
<25 years	459	13.7%	22.9%
25-29 years	987	29.5%	28.4%
30-34 years	1130	33.7%	28.9%
35-39 years	646	19.3%	16.4%
40+ years	129	3.8%	3.5%
Highest level of education			
No formal qualifications	40	1.2%	-
Year 10 or equivalent^#^	301	8.7%	-
Year 12 or equivalent^^^	679	19.6%	-
Trade/Apprenticeship/Certificate/Diploma	1023	29.5%	-
University degree/Postgraduate degree	1423	41.1%	-
Remoteness of residence			
Major city	2221	63.6%	59.4%
Inner regional	671	19.2%	20.4%
Outer regional	485	13.9%	16.0%
Remote and very remote	88	2.5%	3.1%
Not applicable/Outside Queensland	29	0.8%	1.0%
Aboriginal and Torres Strait Islander identification			
None	3390	98.1%	94.2%
Aboriginal and/or Torres Strait Islander	67	1.9%	5.8%
Country of birth			
Australia	2766	79.7%	77.4%
Other country	705	20.3%	22.6%
Parity			
Primiparous	1576	45.3%	40.8%
Multiparous	1906	54.7%	59.2%
Birth plurality			
Singleton	3406	96.5%	98.4%
Multiple	124	3.5%	1.6%
Place of birth			
Public hospital	1916	55.1%	68.0%
Private hospital	1460	42.0%	30.1%
Public birth centre	68	2.0%	1.1%
Home (Planned)	22	0.6%	0.1%
Other	13	0.4%	0.7%

*Note*. Frequencies may not sum to the total due to occasional cases of missing data. *Data were for the entire birthing population in Queensland, Australia in 2010 [23]. ^#^The compulsory level of secondary (high) school required in Queensland. ^^^The final year of secondary (high) school in Queensland.

The sample was approximately representative of the Queensland birthing population on remoteness of residence, country of birth, and parity (see Table 2). The sample under-represented women who were aged less than 25 years, who identified as Aboriginal or Torres Strait Islander, and who reported an ‘other’ place of birth. The sample over-represented women who had a multiple birth and those who birthed in a private hospital, in a birth centre and at home. Sample representativeness on the dimension of education could not be assessed, as these data are not routinely collected for this population.

### Procedure-specific sample sizes

The number of women that reported each of the procedures of interest varied from 424 (for episiotomy) to 3,486 (for ultrasound scan; see Table 3). For all procedures, the size of the sample that reported the procedure was considered sufficient for the planned analyses.

**Table 3:** Number of women reporting each procedure

	**Experienced**	**Not experienced**
**Procedure**	**All women (n = 3530)**	
Ultrasound scan(s)	3,485 (99.6%)	13 (0.4%)
Blood test(s)	3,477 (99.6%)	15 (0.4%)
Induction of labour	870 (24.9%)	2,627 (75.1%)
Pre-labour caesarean section	731 (20.9%)	2,764 (79.1%)
	**Women who had a labour (n = 2764)**	
Vaginal examination(s)	2,432 (92.6%)	195 (7.4%)
Fetal monitoring	2,497 (94.7%)	139 (5.3%)
Post-labour caesarean section	527 (19.1%)	2,232 (80.9%)
Epidural anaesthesia	1,042 (38.3%)	1,676 (61.7%)
	**Women who had a vaginal birth (n = 2237)**	
Episiotomy	424 (19.1%)	1,798 (80.9%)

*Note*. Frequencies may not sum to the total due to occasional cases of missing data.

### Prevalence of decision-making approaches

The proportion of women that reported that they were informed of the benefits and risks of a procedure they experienced ranged widely, from 40% to 96%. Thus, between 4% (for pre-labour caesarean section) and 60% (for vaginal examination(s)) of women reported that they were not informed of the benefits and risks of a procedure they experienced.

The proportion of women that reported being at least consulted in decision-making about a procedure they experienced ranged from 66% to 98%. Thus, between 2% (for epidural analgesia) and 34% (for episiotomy) of women reported that they were unconsulted in decision-making about a procedure they experienced.

At best, 88.2% of women (for epidural analgesia) and 93.5% (for pre-labour cesarean section) reported being both informed of the benefits and risks of the procedure and at least consulted in decision-making. Alternatively, for episiotomy, over one-quarter (26%) of the women who experienced the procedure reported being neither informed nor consulted in decision-making. The same was also true for 18.8% of women who experienced fetal monitoring (see Table 4).

**Table 4:** Prevalence of decision-making approaches by procedure

	**Informed**			**Uninformed**		
**Procedure**	**Decided**	**Consulted**	**Unconsulted**	**Decided**	**Consulted**	**Unconsulted**
Ultrasound scan	936 (27.2%)	805 (23.4%)	41 (1.2%)	586 (17.0%)	754 (21.9%)	320 (9.3%)
Blood test	770 (22.4%)	1171 (34.1%)	57 (1.7%)	303 (8.8%)	713 (20.7%)	423 (12.3%)
Induction of labour	232 (27.1%)	440 (51.3%)	20 (2.3%)	24 (2.8%)	68 (7.9%)	73 (8.5%)
Pre-labour caesarean	350 (48.3%)	328 (45.2%)	21 (2.9%)	10 (1.4%)	11 (1.5%)	5 (0.7%)
Vaginal examination	284 (11.8%)	661 (27.4%)	18 (0.7%)	179 (7.4%)	955 (39.6%)	314 (13.0%)
Fetal monitoring	220 (8.9%)	1,124 (45.5%)	214 (8.7%)	33 (1.3%)	417 (16.9%)	464 (18.8%)
Post-labour caesarean	127 (24.5%)	308 (59.5%)	27 (5.2%)	12 (2.3%)	27 (5.2%)	17 (3.3%)
Epidural analgesia	677 (69.5%)	182 (18.7%)	7 (0.7%)	73 (7.5%)	23 (2.4%)	12 (1.2%)
Episiotomy	33 (7.8%)	175 (41.6%)	34 (8.1%)	2 (0.5%)	66 (15.7%)	111 (26.4%)

*Note*. Frequencies here may not sum to the totals provided in Table 2 due to occasional cases of missing data.

## Discussion and conclusions

Our objective in this study was to examine the extent to which women in Queensland, Australia reported being informed and involved in decisions about the procedures they had during pregnancy, labour, and birth. Consistent with the findings of a similar study in a non-maternity care context in the United States [24], we found considerable variability across the procedures in the prevalence of different decision-making processes. Still, the proportion of women who reported being provided with information and at least consulted in decision-making was considerably smaller than optimal for several procedures (e.g., ultrasound scans, blood tests, vaginal examinations, fetal monitoring, and episiotomy).

Reported patterns in the prevalence of information provision and consumer involvement in decision-making across the procedures allow us to speculate about the factors that may facilitate or impede consumer information provision and involvement in decision-making. While we cannot assess this empirically with available data, we suggest that the prevalence of information provision and consumer involvement may be most proximally determined by the perceived ‘preference sensitivity’ of each procedure, that is, the differential extent to which both having the procedure and not having the procedure are considered clinically reasonable. In turn, beliefs about preference-sensitivity likely affect (and/or are affected by) how embedded a procedure is in routine care, whether there are institutional or clinical policies and guidelines that recommended its use, whether there are tools available to providers to support information provision and consumer involvement in decision-making about the procedure, and whether there are established processes for ensuring and documenting informed consent to the procedure.

The procedure for which the prevalence of the ‘informed decided’ decision-making approach was highest was epidural anaesthesia. Performance of this procedure is typically regarded as at the discretion of the patient [25] and written informed consent is routinely sought. Additionally, excepting episiotomy, procedures performed almost universally (i.e., ultrasound scans, blood tests, fetal monitoring, vaginal examinations) had the highest prevalence of the ‘uninformed unconsulted’ decision-making approach, suggesting that incorporating procedures into routine care may suppress processes of patient involvement.

Patient involvement in decision-making is advocated most strongly, or at times *only*, for decisions that are considered preference-sensitive [26]. However, in maternity care, there is persisting disagreement about the strength of evidence in support of different perinatal procedures, particularly between different sub-groups of providers and stakeholders. This disagreement is reflected in varied perspectives on the preference sensitivity of different perinatal procedures and, accordingly, we were liberal in our selection of procedures to study. Leaving aside differences of opinion about the appropriateness of consumer preferences driving decision-making about some of the procedures examined here, in most situations, there remain legal and ethical obligations for providers to elicit conscious patients’ informed consent to invasive procedures. These obligations seem unlikely to have been fulfilled for the 314 women (13%) in this study who reported being uninformed and unconsulted about the vaginal examinations they experienced and the 111 women (26%) who reported being uninformed and unconsulted about their episiotomies.

It is worthy to note that our approach prioritised women’s subjective perceptions of being informed and involved in decisions about the perinatal procedures they experienced. We regard these subjective perceptions as legitimate and valuable in their own right, and complementary to, rather than inferior proxies of, observational measures of decision-making processes. Put simply, we see little value in the achievement of information provision and patient involvement as judged against an external standard if patients do not simultaneously perceive that they were informed and involved. Nonetheless, these data should not be taken to represent observationally assessed levels of information provision and consumer involvement in decision-making in maternity care. On the basis of previous findings that patients are typically more liberal than third-party observers in their assessments of providers’ shared decision-making behaviours [27-29], we speculate that, if anything, the prevalence of information provision and consumer involvement perceived by women and reported here is inflated. However, without data to confirm or refute this speculation, we are unable to draw any firm conclusions.

A number of study limitations warrant discussion. First, the generalisability of these findings may be impaired by the moderate survey response rate. While this response rate was lower than for similar Australian surveys that have integrated recruitment with health service provision [30,31], our choice to remain independent of the facilities in which these women received care was motivated by our desire to minimise the possible impact of ‘gratitude bias’ [32] on the validity of the data. Moreover, it is important to note that the groups most significantly under-represented within our respondent sample (e.g., younger patients, patients from a minority ethnicity or cultural group) have previously been found to have less self-reported involvement in health-related decision-making [22,33], suggesting that this limitation is likely to have resulted only in us over-estimating the true population prevalence of self-reported information receipt and consumer involvement in decision-making.

Second, the population-level approach we adopted necessitated crude measurement of potentially complex decision-making processes that may occur across multiple providers and/or multiple time points [21]. However, in defending this approach, we consider the inherent costs of simplified assessment at the population level to be balanced by the benefits of large-scale data collection and, particularly, its conduciveness to capturing the perspectives of many diverse individuals, including those frequently unconsulted in research.

Third, the validity of these findings relies on the accuracy of participants’ recall of subjectively experienced decision-making processes, the required duration of which was significant (i.e., up to one year) and varied across some procedures. Although this recall period is considerably shorter than in other studies [24,34], and while consumers typically recall their maternity care experiences with considerable accuracy even years later [35-37], we nonetheless recommend consideration of this when interpreting our findings. We also recommend that complementary research exploring women’s real-time experiences of decision-making be prioritised. Finally, this study represents the first time that the Control Preferences Scale [16] has been adapted in this way. While we conducted cognitive interviews with several women to maximise understandability and validity of these items prior to their use, confidence in our findings would be reinforced by further examination of item performance.

Notwithstanding the limitations noted above, this study provides new and valuable evidence of the current state of decision-making for common perinatal procedures in Australia, as perceived by maternity care consumers. Some findings, including those pertaining to the prevalence of the ‘uninformed unconsulted’ decision-making approach, were especially concerning. These findings highlight the urgent need for interventions that can effectively facilitate information provision and consumer involvement in decision-making, especially for procedures that are considered routine and those within the time-limited episode of intrapartum care. Several studies that have demonstrated the feasibility of sharing decisions with patients in the emergency room [38] confirm that it is not unreasonable to pursue this goal. Moreover, in the maternity care context, most consumers utilise health care frequently in the months preceding birth. This context offers unique opportunities for implementing preparatory strategies that equip consumers with knowledge and skills that allow them participate meaningfully in later intrapartum decision-making, further supporting the feasibility of this goal.

## Competing interests

The authors declare that they have no financial conflicts of interest. The authors acknowledge that this work is based on a fundamental philosophical position that respects and prioritises patients’ right to autonomy and self-determination.

## Authors’ contributions

RT and YM conceived and designed the study and collected data. RT conducted the statistical analysis and RT and YM interpreted the findings. RT drafted the manuscript and YM revised it critically for intellectual content. Both authors read and approved the final manuscript.

## Pre-publication history

The pre-publication history for this paper can be accessed here:

http://www.biomedcentral.com/1471-2393/14/62/prepub
